# Generation of universal natural killer cells from a cryopreserved cord blood mononuclear cell‐derived induced pluripotent stem cell library

**DOI:** 10.1002/2211-5463.13460

**Published:** 2022-07-01

**Authors:** Wei Du, Lijuan Cui, Jinmei Zhang, Hua Zhang, Rongzhi Liu, Wenling Yang, Yu Zhang

**Affiliations:** ^1^ Union Stem Cell & Gene Engineering Co., LTD Tianjin China; ^2^ School of Medicine Nankai University Tianjin China; ^3^ Tianjin Key Laboratory of Blood Cell Therapy Technology China; ^4^ National Stem Cell Product Industrialization Base State Industrial Base for Stem Cell Engineering Products Tianjin China; ^5^ Vcanbio Cell & Gene Engineering Co., Ltd Tianjin China

**Keywords:** cell therapy, cryopreserved cord blood mononuclear cells, induced pluripotent stem cells, natural killer cells

## Abstract

Natural killer (NK) cells play a key role in innate immunity and are regarded as a promising candidate for cellular immunotherapy. Natural killer cells may be generated from different sources, including induced pluripotent stem cells (iPSCs); these stem cells produce an abundant amount of NK cells to meet the needs of a wide range of clinical applications. Autologous iPSCs are expensive and labor‐intensive to prepare, while allogeneic iPSCs require human leukocyte antigen (HLA) matched cells to avoid the risk of immune rejection. In the current study, we prepared HLA‐matched iPSCs using HLA common haplotype homozygous (HLAh) donors from cryopreserved human cord blood (CB) sourced from the Tianjin Cord Blood Public Bank. This approach was designed to generate a CB‐derived iPSC library from HLAh donors and use it to produce off‐the‐shelf NK cells. Starting with readily available cryopreserved CB mononuclear cells (cryoCBMCs), we produced cryoCBMC‐derived iPSCs (cryoCB‐iPSCs). These cryoCB‐iPSCs were induced to generate embryoid bodies (EBs) using an improved 3D suspension culture method, and induced NK (iNK) cells were differentiated from EBs. iNK cells expressed specific surface markers of NK cells, exhibited cytotoxicity comparable with NK cells generated from CB (CB‐NK) and peripheral blood (PB‐NK), and expressed lower levels of KIRs and HLA‐DR compared to CB‐NK and PB‐NK. Taken together, we have shown that an iPSC library can be established from HLAh cryoCBMCs, and cryoCB‐iPSCs can be used to generate a large number of ‘universal’ NK cells for future clinical applications.

## Graphical abstract

Natural killer (NK) cells are a key component of innate immunity as well as adaptive immune cell therapies. Induced pluripotent stem cells (iPSCs) offer an unlimited source for NK derivation. This study shows that an iPSC library can be established from HLA common haplotype homozygous (HLAh) cryopreserved cord blood (cryoCB), and cryoCB‐iPSCs can be used to generate a large number of ‘universal’ iNK cells for future clinical applications.

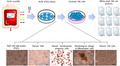

**Abbreviations**
CAR‐T: chimeric antigen receptor T
CB: cord blood
CB‐NK: NK cells generated from CB
cryoCB‐iPSCs: cryoCBMCs‐derived iPSCs
cryoCBMCs: cryopreserved CB mononuclear cells
EBs: embryoid bodies
HLA: human leukocyte antigen
iNK: induced NK
iPSCs: induced pluripotent stem cells
MHC: major histocompatibility complex
NK cells: natural killer cells
PB‐NK: NK cells generated from peripheral blood

There have been many recent developments in immune‐cellular therapy targeting cancer, notably in the field of *chimeric antigen receptor T* (CAR‐T) cell therapy. To date, five CAR‐T products have been approved by the Food and Drug Administration and two CAR‐T products have also been recently launched in China [1]. Natural killer (NK) cells, a unique lymphocyte subgroup, are an important participant of innate immunity and exhibit the capacity to kill virally infected cells and tumor cells. In addition, NK cells exhibit potential value in tumor immunotherapy due to their non‐major histocompatibility complex (MHC) restriction and extensive tumor recognition ability [2]. Natural killer cells can recognize and lyse mutated cells with downregulated MHC‐I molecules or overexpressed activated NK cell receptors [3, 4]. Thus, NK cells, not only in autologous but also allogenetic, can be used to treat cancer without causing serious adverse effects, such as graft‐versus‐host disease (GvHD), holding promise for significantly broadening the availability of clinical application [5].

At present, there are many clinical studies on the applications of NK cells in tumor therapy [3, 5, 6, 7]. The clinical application of NK cells usually requires large and repeated doses to achieve therapeutic effects [8, 9]. Thus, identifying sources that can be used to mass‐produce NK cells to meet clinical requirements has been an active area of research. Indeed, various sources have been tested to generate NK cells, including NK cell lines, peripheral blood‐derived NK cells (PB‐NK), and cord blood‐derived NK cells (CB‐NK) [5, 10]. Natural killer cell lines are unstable and require irradiation before infusion, and the irradiated cells only survive for 48 h [11]. Due to this limitation, NK cell lines cannot achieve long‐term therapeutic effects [12]. PB‐NK and CB‐NK cells are limited for individual dosing, and generating PB‐NK and CB‐NK cells is time‐consuming and cost‐inefficient [10]. Therefore, there is an urgent need to identify an appropriate source to produce a large number of allogeneic NK cells.

Induced pluripotent stem cells (iPSCs) are often generated by the forced expression of relevant pluripotent transcription factors in somatic cells [13]. iPSCs have strong self‐renewal ability and exhibit embryonic stem cell (ES)‐like pluripotency to differentiate into various functional cell lineages [14]. More interestingly, iPSCs have been reported to have the potential to become standard raw materials to produce a large number of NK cells [15, 16]. Further, human CB represents an attractive source of cells for reprogramming iPSCs [17] due to its wide availability, non‐invasive acquisition, low risk of viral contamination and mutation, and it is typically already stored in liquid nitrogen for transplantation. Moreover, the most frequent HLA haplotype homozygous (HLAh) CB selected from public CB bank is an optimal source to produce ‘universal’ iPSCs for a wider range of recipients [18].

In the present study, we designed a strategy to use cryopreserved CB mononuclear cells (cryoCBMCs) to generate iPSCs (cryoCB‐iPSCs). We then improved a method to mass‐produce induced NK (iNK) cells derived from cryoCB‐iPSCs. We characterized iNK cells by evaluating the expression of surface markers of NK cells and cytotoxicity.

## Materials and methods

### CryoCB and cryoCBMC isolation

Human cryoCB was obtained from the Tianjin Cord Blood Public Bank. The donors involved in the study were informed and signed written informed consents. This study was conducted in accordance with the Declaration of Helsinki for experiments involving humans and was approved by the ethical advisory board of the Institute of Hematology and Blood Diseases Hospital (YW2018001‐EC‐1). The CBMC and cryoCBMCs were prepared from fresh and cryopreserved CB, respectively, by Ficoll–Hypaque (G&E Healthcare, Chicago, IL, USA; 17‐1440‐02) as previously described [19].

### Episomal vectors

Oct4‐e2a‐Sox2 (OS), MYC (M), KLF4 (K), and Bcl‐XL (B) were inserted into an EV plasmid backbone, containing Spleen focus‐forming virus U3 (SFFV) promoter, Post‐transcriptional regulatory element (Wpre), Polyadenylation signal from SV40 virus (SV40PolyA), EBV origin of replication (oriP), and Epstein–Barr nuclear antigen 1 (EBNA1) elements as described previously [20]. All insertions of the cloned vectors were verified by sequencing.

### Reprogramming of cryoCBMC to pluripotency

The cryoCBMCs were cultured for 6 days in an erythroid medium as previously described [19]. The 4 × 10^5^ cells were nucleofected with a 1 μL plasmid mixture (400 ng·μL^−1^ OS, 200 ng·μL^−1^·M, 200 ng·μL^−1^ K, and 100 ng·μL^−1^ B), and distributed to one well in a vitronectin‐treated 6‐well plate and cultured for another 2 days. After 2 days, the culture medium was changed to the induction medium (2 mL·well^−1^), which contained Knockout™ DMEM/F12 (Gibco, Grand Island, NY, USA; 12660012) with 50 ng·mL^−1^ basic fibroblast growth factor (Peprotech, Rocky Hill, NJ, USA; 100‐18C‐10), 13 ng·mL^−1^ Insulin‐Transferrin‐Selenium (Gibco; 41400‐045), 2 mm l‐glutamine (Gibco; 25030081), and 50 mg·mL^−1^ ascorbic acid (Sigma, St. Louis, MO, USA; 49752‐10G). The induction medium was replenished every 2 days, and on day 6, was changed to another induction medium that contained 0.25 mm sodium butyrate (Sigma; B5887‐250MG), which was replenished every 2 days until day 12. The resulting iPSCs were cultured *in vitro* nectin‐treated 6‐well plates and refreshed with mTeSR1 medium (Stemcell, Vancouver, BC, Canada; 85850) every 2 days for long‐term culture.

### Alkaline phosphate staining and immunocytochemistry

Alkaline phosphatase (AP) staining was performed using an Alkaline Phosphatase Staining Kit II (Stemgent, Cambridge, MA, USA; 00‐0055). For detection of pluripotent stem cell marker antigens, cells were fixed with 4% paraformaldehyde in PBS for 10 min at room temperature. After washing with PBS, cells were incubated in 0.25% Triton X‐100 (Sigma‐Aldrich; X100‐1L) for 10 min at room temperature, and then blocked with 5% sheep or donkey serum for 30 min at room temperature. Cells were then incubated with the following primary antibodies SSEA‐4 (1 : 100; Invitrogen, Carlsbad, CA, USA; MA1‐021X), TRA‐1‐60 (1 : 50; Invitrogen; MA1‐023), Oct‐4 (1 : 100; Invitrogen; PA5‐27438), SOX2 (1 : 100; Invitrogen; PA1‐094X), and Nanog (1 : 100; Invitrogen; 14‐5768‐82), overnight at 4 °C. Cells were then incubated with appropriate secondary antibodies corresponding to the primary antibody at room temperature for 1 h. The secondary antibodies included Alexa Fluor 594 AffiniPure Donkey anti‐mouse IgG (1 : 250; Jackson, Bar Harbor, ME, USA; 715‐585‐150), Alexa Fluor 488 AffiniPure Donkey anti‐rabbit IgG (1 : 250; Jackson; 711‐545‐152) and Alexa Fluor 594 AffiniPure goat anti‐mouse anti‐mouse IgG (1 : 250; Jackson; 115‐585‐075). The nuclei were stained with DAPI (1 : 10; Vector Laboratories, Burlingame, CA, USA; H‐1200).

### Differentiation capacity of cryoCB‐iPSCs *in vitro*

STEMdiff™ Trilineage Differentiation Kit (Stemcell; 5230) was used to differentiate cryoCB‐iPSCs into endoderm, mesoderm, and ectoderm. Mesoderm and endoderm lineages were formed on day 5, and ectoderm lineages were formed on day 7. Immunofluorescence staining was used to detect representative markers (AFP3 for mesoderm, 1A4 for endoderm and 10C2 for ectoderm) of cryoCB‐iPSCs. The Human Pluripotent Stem Cell Trilineage Differentiation quantitative polymerase chain reaction (qPCR) Array (Stemcell; 7515) was performed to characterize hPSCs and their trilineage differentiation capacity. This qPCR array was designed to detect the gene expression profile of undifferentiated hPSCs and their trilineage derivatives following in vitro directed or spontaneous differentiation. Genes were selected based on their demonstrated differential expression in ES and iPSCs compared with hPSC‐derived ectodermal, mesodermal, and endodermal lineage cells.

### Teratoma formation assay and histological analysis

Approximately 1 × 10^6^ cryoCB‐iPSC after 15 passages in culture were harvested with Accutase (Stemcell; 07920) and resuspended in 200 μL solution that was prepared with Matrigel (Corning, Acton, MA, USA; 354277) and DMEM/F12 medium (HyClone, Logan, UT, USA; SH30243.01) at a ratio of 1 : 1. These cells were subcutaneously injected into the rear haunch of each NOD/SCID immunodeficient mouse. Two months after implantation, the formed teratomas were removed and sectioned at 7 μm thickness for hematoxylin and eosin (H&E) staining.

### NK cells induced from cryoCB‐iPSCs

**Fig. 1:**
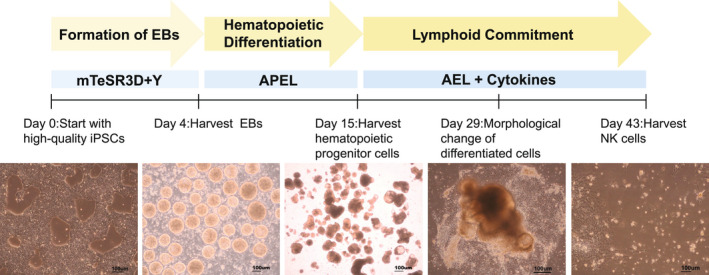
Schematic representation showing a three‐stage protocol for the generation of NK cells from iPSCs.

To produce NK cells from cryoCB‐iPSCs, we developed a method for EB formation from 3D cultured iPSCs. In brief, cryoCB‐iPSCs (8 × 10^5^ cells·mL^−1^) were transferred and cultured in non‐tissue cultured (TC) treated six‐well plates (2 mL mTeSR™ 3D Medium (Stemcell; 03950) per well). Cells were incubated on a shaking table with a rotation speed of 70 r.p.m. at 37 °C, 5% CO_2_ for 4 days. Morphological changes of cryoCB‐iPSCs were monitored every day. On the fourth day, EBs were approximately spherical with a diameter of 150–250 nm. EBs were then transferred to non‐TC treated six‐well plates at a density of 300–400 per well. Then the classic two‐step differentiation method was used to induce NK (iNK) cells as previously described [16, 21]. Single cells in suspension culture were filtered out. EBs were transferred in differentiation medium (APEL™ medium (Stemcell; 05275) and PFHM‐II at a proportion of 20 : 1, including VEGF (20 ng·mL^−1^) and BMP4 (20 ng·mL^−1^), SCF (40 ng·mL^−1^)), the density was adjusted to 40–60 EBs per well. EBs were then incubated at 37 °C in a 5% CO_2_ incubator for 11 days. Blastocyst‐like EBs were generated and the APEL differentiation medium was replaced with an AEL differentiation medium (including IL‐3 (5 ng·mL^−1^), IL‐15(10 ng·mL^−1^), FLT3(10 ng·mL^−1^), IL‐7(20 ng·mL^−1^), and SCF (20 ng·mL^−1^)), which led to lymphocyte differentiation. AEL differentiation medium was replaced every week. After 4 weeks, iNK cells were harvested in suspension (Fig. 1).

### Flow cytometry

To investigate phenotypic changes during differentiation of cryoCB‐iPSCs, FACSCalibur flow cytometry (BD Biosciences, San Diego, CA, USA) was used to characterize cryoCB‐iPSCs with antibodies against CD34 (catalog no. Z6410008), CD45 (catalog no. Z6410006), CD3 (catalog no. Z6410026), and CD16 (catalog no. Z6410070; Quantobio, Beijing, China); CD56 (catalog no. 562794), NKp46 (catalog no. 557991), NKp44 (catalog no. 558563), NKpG2D (catalog no. 562064), and CD94 (catalog no. 559876; BD Biosciences). Isotype controls were used to eliminate background due to the nonspecific binding of antibodies to cell surfaces.

### Cytotoxicity assay of iNK cells

CD107a expression and intracellular IFN‐γ and TNF‐α production determined by flow cytometry were used to evaluate the cytotoxicity of iNK cells. In brief, iNK cells (i.e., effector cells) were co‐cultured with K562 cells (i.e., target cells) at a ratio of 1 : 1. iNK cells that were not co‐cultured with target cells were used as a negative control. iNK cells were collected after 5 h incubation and stained with CD56‐FITC (catalog no. 562794), CD107a‐APC (catalog no.560664), IFN‐γ‐PE (catalog no. 559327; BD Biosciences), and TNF‐α‐APC (catalog no. 502912; Biolegend, San Diego, CA, USA). The expression of CD107A, IFN‐γ, and TNF‐α was analyzed and compared in iNK cells between the two groups.

To detect direct cytotoxicity of NK cells against target cells, a flow cytometry‐based method was used [10, 12]. In brief, 0.5 × 10^5^ to 5 × 10^5^ NK cells were cocultured with 5 × 10^4^ carboxyfluorescein diacetate succinimidyl ester (CFSE) – labeled cancer cells at various effector to target (E : T) ratios for 4 h. Samples were then stained on ice with 7‐AAD for 10 min. After washing, target cell death was assessed with a flow cytometer by the percentage of 7‐AAD‐stained cells in the CFSE‐positive population. To inhibit the activity of the perforin/granzyme system in NK cells, the co‐culture killing assays were performed in the presence of 5/3 mm EGTA/Mg^++^. Target cells (CFSE^+^) were gated, and the percent of 7‐AAD^+^ cells was used to calculate NK cell cytotoxicity using the following equation: (Experimental–Spontaneous dead cells/(100–Spontaneous dead cells) × 100%.

### Statistical analysis

All analyses were performed with spss 25.0 software (SPSS Inc., Chicago, IL, USA). Data were expressed as mean ± standard error of the mean (SEM). ANOVA was used for the comparison of variables among multiple groups. Student *t*‐test was used for comparison of variables between two groups. A *P* value < 0.05 was considered statistically significant.

## Results

### Characterization of cryoCB‐iPSCs

**Fig. 2:**
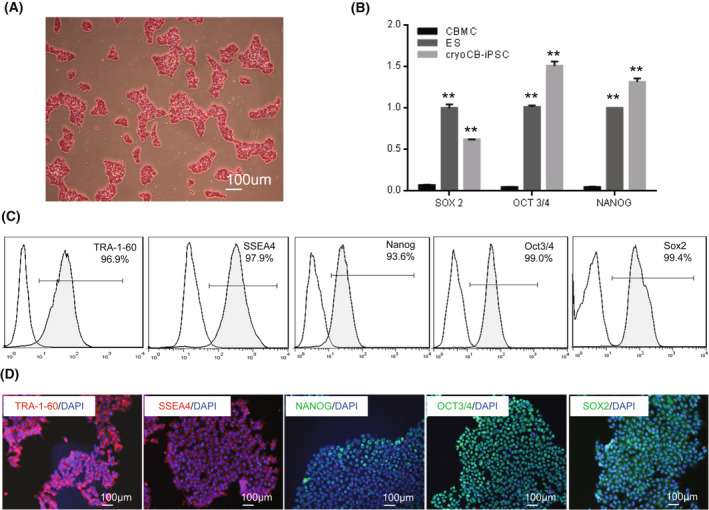
Characterization of cryoCB‐iPSCs. (A) Alkaline phosphatase staining of cryoCB‐iPSCs. Scale bar, 100 μm. (B) Comparison of expression of pluripotency gene, SOX2, OCT3/4, and NANOG, between cryoCB‐iPSCs and CBMC‐iPSCs and H1 human ES cells (*n* = 5). Data are represented as the mean ± SEM. **P < 0.01 vs. CBMC as determined by Student *t*‐test. (C) Flow cytometry analysis of the expression of pluripotency markers, TRA‐1‐60, SSEA4, NANOG, OCT3/4, and SOX2, on cryoCB‐iPSCs. (D) Representative images of immunofluorescence staining showing expression of typical ES cells markers TRA‐1‐60 (red), SSEA4 (red), NANOG (green), OCT3/4 (green), and SOX2 (green) on cryoCB‐iPSCs. Nuclei are stained with DAPI (blue). Scale bar, 100 μm.

The cryoCB‐iPSCs were positive for AP staining (Fig. 2A). The expression of pluripotency markers SOX2, OCT3/4, and NANOG were markedly increased in the cryoCB‐iPSCs compared with the parental CBMCs, and were comparable to expression in H1 human ES cells as revealed by RT‐qPCR (Fig. 2B). Also, pluripotency markers TRA‐1‐60, SSEA4, NANOG, OCT3/4, and SOX2 were markedly expressed in the cryoCB‐iPSCs as revealed by flow cytometry (Fig. 2C). Consistent with the above observations, immunofluorescence staining showed that the cryoCB‐iPSCs were positive for typical ES cell markers such as TRA‐1‐60, SSEA4, NANOG, OCT3/4, and SOX2 (Fig. 2D).

### Differentiation of cryoCB‐iPSCs

**Fig. 3:**
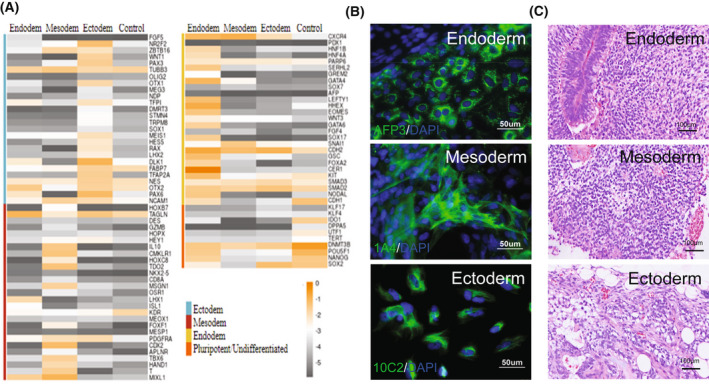
Differentiation capability of cryoCB‐iPSCs. (A) Trilineage differentiation quantitative PCR array of cryoCB‐iPSCs. (B) Representative images of immunofluorescence staining of markers of different lineage cells: AFP3 for mesoderm, 1A4 for endoderm, and 10C2 for ectoderm. Scale bar, 50 μm. (C) Representative images of H&E staining of teratomas containing endoderm, mesoderm, and ectoderm. Scale bar, 100 mm.

We next examined the differentiation capability of cryoCB‐iPSCs using hPSC Trilineage Differentiation qPCR Array, and found that cryoCB‐iPSCs could differentiate into ectoderm, mesoderm, and endoderm cells (Fig. 3A). Immunofluorescence staining showed that representative markers, AFP3, 1A4, and 10C2, were positive for mesoderm, endoderm, and ectoderm, respectively (Fig. 3B). We also subcutaneously injected cryoCB‐iPSCs into immunosuppressed mice and examined the teratoma formation after 2 months by H&E staining. H&E staining showed that the teratoma contained three germ layer tissues (Fig. 3C).

### Safety evaluation of cryoCB‐iPSCs

**Fig. 4:**
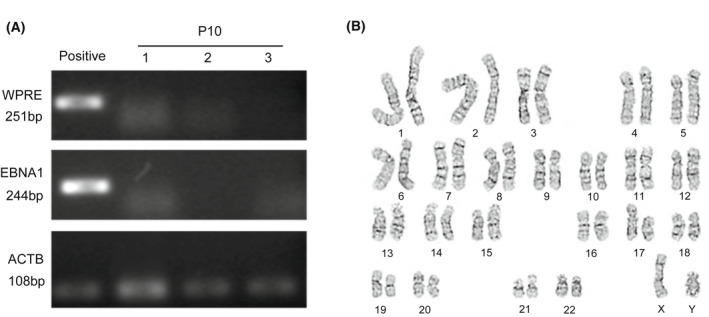
Safety evaluation of cryoCB‐iPSCs. (A) PCR‐based detection of vector sequence (EBNA1 and WPRE) was not found in the expanded cryoCB‐iPSCs after 10 passages. (B) A representative karyotyping image of a cryoCB‐iPSC.

We selected three cryoCB‐iPSCs and analyzed the changes in average copies of total plasmids. Zero copies of the plasmid were detected in cells from passage 10 of the three cryoCB‐iPSCs (Fig. 4A), suggesting that plasmids were depleted from these cells. Karyotype analysis was performed to evaluate the genomic stability of cryoCB‐iPSCs at passage 15, and showed a normal karyotype (Fig. 4B), suggesting that long‐term cultured cryoCB‐iPSCs did not exhibit detectable chromosomal abnormalities.

### HLAh CB in Tianjin Cord Blood Public Bank

To demonstrate the feasibility of generating a CB‐derived HLAh iPSC library, we investigated HLAh CB from the Tianjin Cord Blood Public Bank. We found that there were 16 homozygous CBs, including HLA‐A, ‐B, ‐C, ‐DR, and ‐DQ. The genotype frequency of these 16 homozygous CBs was 6.5% in the Chinese population, covering 11.3% of the total Chinese population (Table 1) based on high‐resolution analyses of HLA‐A, ‐B, ‐C, ‐DR, and ‐DQ frequencies of 169 995 volunteers from the China Bone Marrow Donor Registry Program [22].

**Table 1:** Genotypes and frequency of 16 HLAh cord blood from the Tianjin cord blood public bank.

Numbers	Freq. (‰)	HLA‐A		HLA‐B		HLA‐C		HLA‐DR		HLA‐DQ	
10	37.0023	30:01	30:01	13:02	13:02	06:02	06:02	07:01	07:01	02:02	02:02
4	24.6414	02:07	02:07	46:01	46:01	01:02	01:02	09:01	09:01	03:03	03:03
1	5.7905	02:03	02:03	38:02	38:02	07:02	07:02	16:02	16:02	05:02	05:02
1	3.6439	02:01	02:01	39:01	39:01	07:02	07:02	11:06	11:06	03:01	03:01

### Characteristics of iNK Cells

**Fig. 5:**
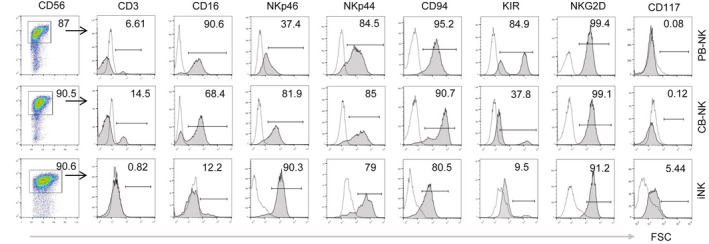
Detection of surface markers, CD56, CD16, Nkp46, NKp44, CD94, KIR, NKG2D, and CD117, on PB‐NK cells, CB‐NK cells, and iNK cells by flow cytometry.

We first characterized iNK cells by examining the expression of surface markers. Compared to PB‐NK and CB‐NK cells, iNK cells had the expression of immunoglobulin‐like receptors (KIRs), CD16, NKp46, NKp44, CD94, NKG2D, and CD117 indicating that iNK cells can be generated from cryoCB‐iPSCs using our EB differentiation method. In addition, we also found that iNK cells expressed lower levels of KIRs and CD16, compared to PB‐NK and CB‐NK cells (Fig. 5).

### Immunogenicity of cryoCB‐iPSC and iNK cells

**Fig. 6:**
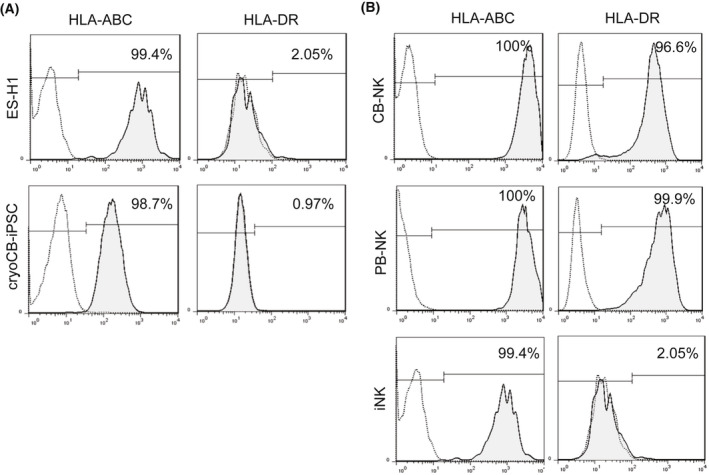
Immunogenicity of cryoCB‐iPSCs and iNK cells. (A) Expression of HLA‐I (A, B, C) and HLA‐II (DR) in cryoCB‐iPSCs and ES‐H1 cells. (B) Expression of HLA‐I (A, B, C) and HLA‐II (DR) in iNK, PB‐NK, and CB‐NK cells.

We next examined the expression of HLA‐I (A, B, C) and HLA‐II (DR) in cryoCB‐iPSCs and iNK cells to assess their immunogenicity. The cryoCB‐iPSCs and ES‐H1 had a comparable expression of HLA‐I (A, B, C) and HLA‐II (DR; Fig. 6A). Compared to CB‐NK and PB‐NK cells, iNK cells expressed comparable levels of HLA‐I (A, B, C) and lower levels of HLA‐II (DR) (Fig. 6B). These results demonstrate that iNK cells have low immunogenicity and may become ‘universal’ NK cells.

### Cytotoxicity of iNK cells against cancer cells

**Fig. 7:**
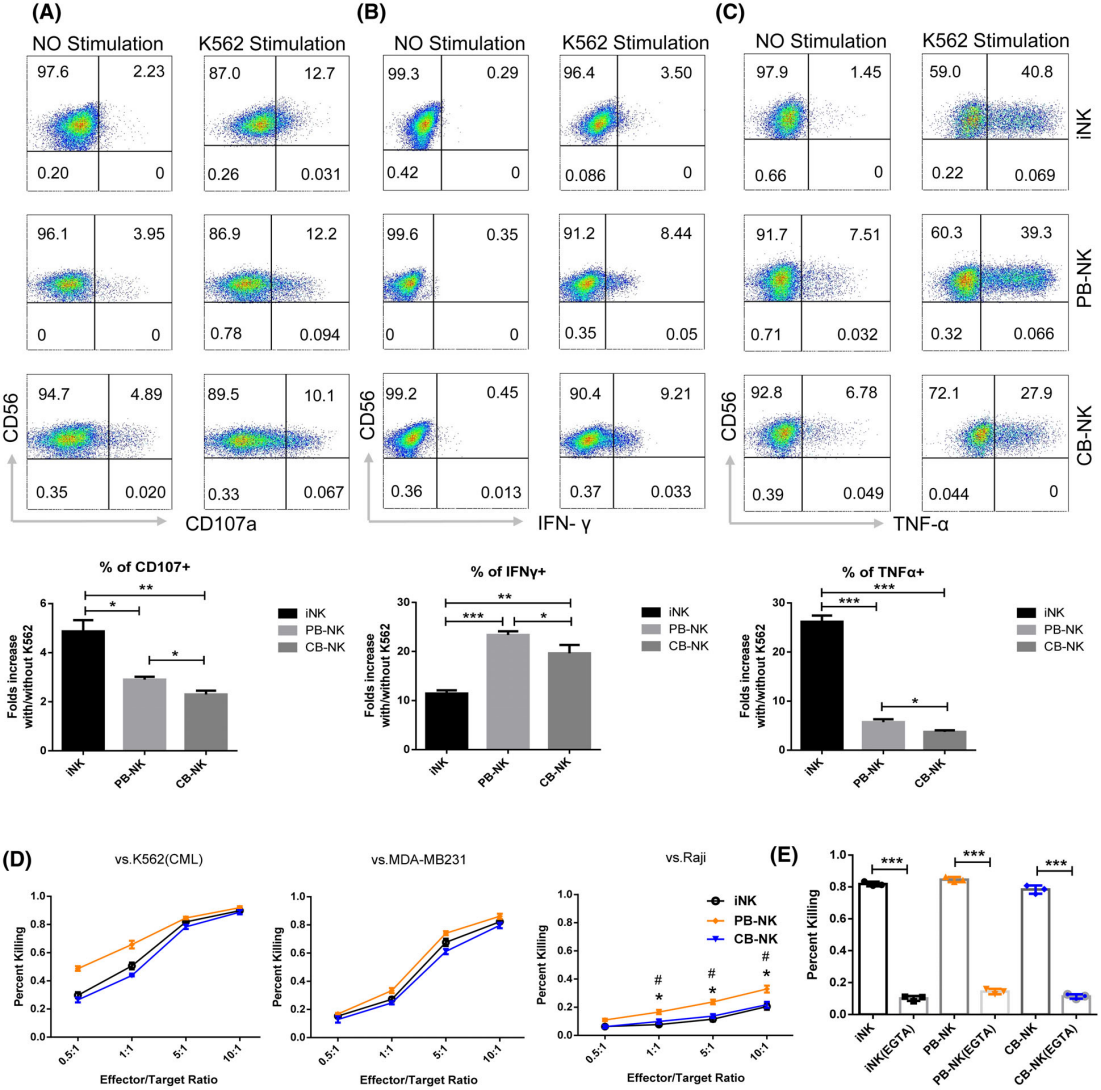
Functional characterization of iNK cells. (A) Expression of CD107a in iNK, PB‐NK, and CB‐NK cells (*n* = 3 for each group). Data are represented as the mean ± SEM. *P < 0.05 and **P < 0.01 as determined by ANOVA. (B) Expression of IFN‐γ in iNK, PB‐NK, and CB‐NK cells (*n* = 3). Data are represented as the mean ± SEM. *P < 0.05, **P < 0.01, and ***P < 0.001 as determined by ANOVA. (C) Expression of TNF‐α in iNK, PB‐NK, and CB‐NK cells (*n* = 3). Data are represented as the mean ± SEM. *P < 0.05 and ***P < 0.001 as determined by ANOVA. (D) Cytotoxicity assay against K562, MDA‐MB‐231, and Raji (*n* = 3). Data are represented as the mean ± SEM. * or #*P* < 0.05; *: PB‐NK vs. iNK; #: PB‐NK vs. CB‐NK as determined by ANOVA. iNK cells killed K562 cells and MDA‐MB‐231 cells with comparable capacity as PB‐NK and CB‐NK cells. PB‐NK cells killed Raji cells significantly better than iNK and CB‐NK cells. (E) Cytotoxicity assay against K562 (effector/target ratio = 5 : 1) in the presence of EGTA. Data are represented as mean ± SEM (*n* = 3). Data are represented as the mean ± SEM. ***P < 0.001 as determined by Student *t*‐test.

We investigated the cytotoxicity of the generated NK cells by co‐culturing three types of NK cells, iNK, PB‐NK, and CB‐NK cells, with K562 cells (chronic myeloid leukemia cell line) at a ratio of 1 : 1, respectively, followed by flow cytometry analysis for staining of CD56‐FITC, CD107a‐APC, and IFN‐γ‐PE. The NK cells cultured without target cells were used as a control. Compared to CB‐NK and PB‐NK cells, iNK cells expressed higher levels of CD107a expression and TNF‐α secretion, and lower levels of IFN‐γ secretion (Fig. 7A–C).

We also evaluated the direct cytotoxicity against cancer cells by assessing cytotoxicity induced by NK cells on K562, MB‐MDA‐231 (breast cancer cell line), and Raji (lymphoma cell line). iNK cells were able to efficiently kill K562 cells and MB‐MDA‐231. However, PB‐NK cells induced the highest toxicity on Raji cells compared with iNK or CB‐NK cells (Fig. 7D). To determine whether NK‐induced cytotoxicity depends on cytotoxic granule release, cytotoxicity was measured in the presence or absence of the Ca^++^ chelator EGTA, which inhibits cytotoxic granule release. EGTA completely blocked cytotoxicity, suggesting that degranulation was required (Fig. 7E).

## Discussion

Natural killer cells have marked potential value in tumor immunotherapy due to characteristics such as non‐MHC restriction and extensive tumor recognition [3, 23]. However, the main problem hindering the clinical application of NK cells is the production of large numbers of NK cells required for therapeutic clinical applications [24, 25]. Therefore, there is an urgent need for a method that can be used to mass‐produce NK cells [5, 25]. To address this unmet need we designed an approach to produce unlimited NK cells from cryoCB‐iPSCs. Using an improved EB formation protocol, functional mature NK cells were produced from cryoCB‐iPSCs. These iNK cells expressed NK cell‐specific surface markers, exhibited cytotoxicity, but had less KIRs and HLA‐DR. Therefore, we believe that this approach may be used to generate a large number of ‘universal’ functional NK cells, enabling wider clinical application of tumor immunotherapy.

Currently, therapeutic NK cells are primarily derived from NK cell lines, PB and CB. In recent years, accumulating evidence has suggested that iPSCs are a promising source of NK cells [13, 26]. For example, iPSC‐derived NK cells have been used in several clinical studies to treat tumors [27]. Further, a large number of preclinical and clinical studies using autologous iPSCs‐derived functional cells have been used to treat various diseases in regenerative medicine [28]. Notably, autologous iPSCs have been used to produce retinal pigment epithelial cells to treat age‐related macular degeneration [29]. Unfortunately generating autologous iPSCs is expensive and labor‐intensive which has limited the therapeutic value of this approach.

An alternative strategy is to provide iPSC products using universal HLAh donors. For this purpose, CB represents an excellent source to produce iPSCs compared with adult cells, such as PBMCs and skin fibroblasts [17]. Previous results have shown that iPSCs derived from adult tissues have higher levels of mitochondrial DNA mutations than those derived from CBMCs [30], suggesting an advantage of using CB cells to generate iPSCs. Moreover, the most common HLA CB can be selected from a CB bank to produce iPSCs for a wider range of applications. As previously reported, high‐resolution HLA CB has been used to establish an iPSCs bank, and the 10 most common homozygous iPSC lines, matching 41.07% of the Korean population, have been characterized [18]. Taylor et al. [31] showed that 14 high‐frequency homozygous iPSC lines could provide HLA‐matched donors for 58.11% of the UK population. We assayed HLAh CB from the Tianjin Cord Blood Public Bank and found that there were 16 homozygous CBs, including HLA‐A, ‐B, ‐C, ‐DR, and ‐DQ. The genotype frequency of the 16 homozygous CB was 6.5% in the Chinese population, matching 11.3% of the Chinese population.

KIRs, also known as CD158, are a group of transmembrane glycoproteins that are universally expressed on NK cells and are key regulators of NK cell cytotoxicity [32, 33]. The levels of KIR expression on NK cells mediate the cytotoxicity of NK cells, and targeting KIRs has been shown to be a therapeutic approach to improve clinical outcomes. In addition, a KIR‐HLA mismatch donor has to be selected for patients to achieve optimal outcomes [34]. Previously, iPSCs derived from PBMCs were shown to generate KIR‐negative NK cells, which may contribute to their clinical efficacy [12]. Consistent with the observations above, in the present study we also found that cryoCB‐iPSCs were able to produce KIRs‐negative iNK cells. In addition to the expression of low levels of KIRs, these iNK cells all expressed typical NK cell surface molecules. Therefore, we hypothesize that our method of using croCB‐iPSCs to generate a large number of iNK cells is a viable alternative to the traditional methods which will enable additional clinical applications.

Inducing differentiation of iPSCs toward NK cells is a key step in the application of NK cells. One method is to produce CD34^+^ hematopoietic progenitor cells from iPSCs, then promote CD34^+^ hematopoietic progenitor cells to differentiate into NK cells [16, 35]. That protocol is complex and reduces the yield of NK cells because of the exclusion of many other hematopoietic progenitors [16, 35]. Another method is to form EBs, collect EBs, and then differentiate EBs into NK cells. The traditional method to generate EBs is to inoculate iPSCs with 3000 cells per well into a 96‐well plate and then rotate it under appropriate conditions [36]. The generated EBs are then collected and distributed every six spins in one well of a 24‐well plate, and differentiated into NK cells. Technically, this method is labor‐intensive and difficult to mass‐produce NK cells for clinical applications [36]. In the present study, we established an improved protocol for EB formation by transferring cryoCB‐iPSCs at a density of 8 × 10^5^ cells·mL^−1^ to non‐TC treated six‐well plates with mTeSR™ 3D Medium. These cells were then incubated on a shaking table with a rotation speed of 70 r.p.m. at 37 °C in a 5% CO_2_ incubator for 4 days. Embryoid bodies were formed with approximately spherical shapes with a diameter of 150^_^250 nm on the fourth day. Embryoid bodies were then transferred to non‐TC treated six‐well plates at a density of 300^_^400 per well for differentiation into hematopoietic cells. This improved protocol is easy for large‐scale production of NK cells, which we believe will meet the needs of various clinical applications.

## Conclusion

We report a technique to generate NK cells, that is, ‘from cryoCB to iPSCs, then back to NK cells’, and characterized the resulting NK cells. We found that these iNK cells were similar to those generated from other sources with regard to surface marker expression and functions such as cytotoxicity. Our work demonstrates that this technique is a viable approach to mass‐producing NK cells for various clinical applications.

## Conflict of interest

The authors declare no conflict of interest.

## Author contributions

Conceptualization, WD and YZ; Funding acquisition, WD and YZ; Methodology, WD, LC, JZ, HZ and RL; Supervision, WY and YZ; Writing – original draft, WD and LC; Writing – review and editing, YZ.

## Data Availability

The authors confirm that all of the data supporting the findings of the present study are available within the article.
